# Risk Factors of Incidental Parathyroidecomy in Thyroid Surgery

**DOI:** 10.7759/cureus.6517

**Published:** 2019-12-30

**Authors:** Thamer Alraddadi, Saleh Aldhahri, Mohammad Almayouf, Jabir Alharbi, Moayyad Malas, Muhammad Nasrullah, Khalid Al-Qahtani

**Affiliations:** 1 Otolaryngology, King Saud University, Riyadh, SAU; 2 Otolaryngology, Head & Neck Surgery, King Saud University, Riyadh, SAU; 3 Otolaryngology, Head & Neck Oncology Surgery, King Fahad Medical City, Riyadh, SAU; 4 Otolaryngology, Majmaah University, Riyadh, SAU; 5 Otolaryngology, King Khaled Hospital, King Abdulaziz Medical City, Jeddah, SAU; 6 Otolaryngology, King Fahad Medical City, Riyadh, SAU

**Keywords:** incidental parathyroidectomy, risk factors, surgical loupes, thyroidectomy

## Abstract

Background: Incidental parathyroidectomy with subsequent hypoparathyroidism and postoperative hypocalcemia is thought to be one of the common complications of thyroidectomy. Current literature reports wide discrepancy in incidence and risk factors.

Objectives: The aim of our study was to evaluate the incidence and risk factors of incidental parathyroidectomy in thyroid surgery.

Methods: A retrospective study included 270 patients who had thyroid surgery that was performed over two years from January 2017 to December 2018 in two tertiary care hospitals. Preoperative and postoperative records were assessed. Factors such as gender, diagnosis, type of surgery, and usage of surgical loupes during the procedure were evaluated and were compared to find the association with incidental parathyroidectomy in thyroid surgery.

Results: Incidental parathyroidectomy was noticed in 62 (23%) surgical specimens during histopathologic examination. There was no significant association between incidental parathyroidectomy and sex of patient, use of surgical loupes, pathology of thyroid disease, or neck dissection.

Conclusion: Although the risk of incidental parathyroidectomy is inevitable, careful dissection and meticulous intraoperative identification of parathyroid gland during thyroidectomy can reduce the incidence of incidental parathyroidectomy, thereby minimizing the risk of postoperative hypoparathyroidism and hypocalcemia.

## Introduction

Thyroidectomy is a common, relatively safe surgical procedure, which is associated with <5% morbidity rate [1]. The main postoperative complications of thyroidectomy include injury to the parathyroid glands and recurrent laryngeal nerves. Because of its small size and close proximity to the thyroid tissue, inadvertent injury to the parathyroid glands by devascularization and accidental resection are inevitable, which most often can result in symptomatic hypoparathyroidism and hypocalcemia [2,3]. Hypocalcemia could either be transient or permanent. While the reported incidence of the transient hypocalcemia ranges between 10% and 46%, the permanent one ranges between 1.5% and 4% [4]. Therefore, it is needless to say that understanding the anatomy of the parathyroid and the ability to identify them will help in avoiding this complication.

Finding a parathyroid gland in postoperative thyroidectomy specimen without any intention to do parathyroidecomy is known as incidental parathyroidectomy (IP). The incidence ranges between 6.4% and 31.1% [5-11]. Over the past decades, standardization of the thyroidectomy technique and advances in perioperative management have led to significant reduction in the overall morbidity [5]. Although IP is considered a minor finding in final histopathology and not a life-threatening complication of thyroid surgery, it is important for the thyroid surgeon to be able to identify factors that increase the risk for IP during thyroidectomy and exercise appropriate caution in those patients [6]. Preoperative diagnosis, type of operation, and presence of nodal metastases may influence the likelihood of IP; however, there is no unanimity of risk factors agreed upon in the literature [7].

The present study was conducted to evaluate the incidence and risk factors of IP in thyroid surgery.

## Materials and methods

A multicenter retrospective study was conducted in two tertiary care hospitals in Riyadh, Saudi Arabia, to evaluate the incidence and risk factors of IP during thyroid surgeries. The duration of study was two years from January 2017 to December 2018. All surgeries were performed by head and neck consultants who had more than 10 years of experience in thyroid surgery. The patient's medical records were used to collect the demographic and medical data for all patients who underwent total thyroidectomy or completion with or without neck dissection. Patients who underwent hemithyroidectomy or thyroidectomy with parathyroidectomy and those with incomplete clinical data were excluded from the study. Factors such as gender, diagnosis, type of surgery, and usage of surgical loupes during the procedure were evaluated. The chi-square test was used to calculate P-value. A P-value less than 0.05 was considered statistically significant. SPSS version 20 software (IBM Corp., Armonk, NY) was used.

## Results

A total of 270 patients were included in the study, with 281 (80.7%) predominantly females and only 52 (19.3%) males. On the basis of final pathology, 50.7% were benign, whereas 34.8% were classical type of papillary thyroid carcinoma, 10% were follicular variants of papillary thyroid carcinoma, 0.7% were insular type of papillary thyroid carcinoma, 1.1% were follicular thyroid carcinoma, Hurthle cell carcinoma seen in one patient as seen in lymphoma and squamous cell carcinoma, and 1.5% were follicular tumor of uncertain malignant potential. Table 1 illustrates the diagnoses of the studied sample.

**Table 1:** Final Pathology Result

Diagnosis	Frequency	Percentage (%)
Benign	137	50.7
Papillary thyroid carcinoma classical type	94	34.8
Papillary thyroid carcinoma follicular variants	27	10
Papillary thyroid carcinoma insular type	2	0.7
Follicular thyroid carcinoma	3	1.1
Hurthle cell carcinoma	1	0.4
Follicular tumor of uncertain malignant potential	4	1.5
Squamous cell carcinoma	1	0.4
Lymphoma	1	0.4

For the type of surgery, 234 (86.7%) patients underwent only total thyroidectomy, 12 (4.4%) patients had completion thyroidectomy, 12 (4.4%) patients had total thyroidectomy with unilateral neck dissection, 9 (3.3%) patients underwent total thyroidectomy with central neck dissection, total thyroidectomy with bilateral neck dissection was performed in two patients, and one patient had completion thyroidectomy with unilateral neck dissection. Table 2 illustrates the type of surgery. Regarding surgical technique, all procedures were performed using identical techniques: making the incision, raising the flap, ligating the superior thyroid vessels, identifying the recurrent laryngeal nerve, and visualizing the the parathyroid gland. However, 65.4% of the cases were operated with the assistant of surgical loupes.

**Table 2:** Type of Surgery Performed and Its Relation with Incidental Parathyroidectomy ANOVA, analysis of variance.

Type of surgery	Incidental parathyroidectomy		P-value
	No	Yes	ANOVA test (P=0.813)
Total thyroidectomy	182	52	
Total thyroidectomy + central neck dissection	4	5	
Total thyroidectomy + unilateral neck dissection	11	1	
Total thyroidectomy + bilateral neck dissection	2	0	
Completion thyroidectomy	8	4	
Completion thyroidectomy + unilateral neck dissection	1	0	

Parathyroid tissue was found in the histopathology specimens of 62 (23%) patients. Comparative analysis showed no significant statistical association between incidence of parathyroidectomy and patient factor (gender), surgery factors (type of surgery), surgeon factors (loupes or without loupes), and tumor factors (malignant or benign). Table 3 shows the factors affecting IP.

**Table 3:** Factors Affecting IP IP, incidental parathyroidectomy.

IP	Gender			P-value
	Male	Female		0.450
No	38	170		
Yes	14	48		
SURGEON				
	With loupes	Without loupes		0.680
No	135	73		
Yes	42	20		
HISTOLOGY				
	Benign	Malignant	Micropapillary thyroid carcinoma	0.512
No	111	58	39	
Yes	30	22	10	

## Discussion

The present study was conducted to evaluate the risk factors of IP. The incidence of IP was 23%, which is comparable with the incidence found in the literature which ranges from 6.4% to 31.1% [8-11]. This suggests that even with excellent knowledge of anatomy and meticulous dissection, the possibility of IP is inevitable. The incidence can be decreased by careful dissection using the capsular separating technique performed by a highly experienced surgeon.

Identification of the glands during surgery may be tricky even with a careful capsular dissection technique due to various locations of parathyroid tissue. Therefore, it is not always possible to identify all parathyroid glands. Hence, even with improvement in surgical techniques, the risk of IP cannot be eliminated. Although the various locations of parathyroid glands make the intraoperative identification a difficult procedure, it is advised by various authors to carefully inspect the specimen for the presence of parathyroid glands and autotransplant them onto an adjacent sternomastoid muscle [12,13].

In the past, researchers have aimed to identify potential risk factors associated with IP. Youssef et al. found concomitant central neck dissection and reoperation for recurrent goiter sustained as risk factors of IP through their multivariate analysis [14]. According to Sippel et al., younger age, malignant pathology, and bilateral thyroid resection existed as risk factors of incidental parathyroid resection [15]. Risk factors for IP such as female sex, diagnosis of thyroid disease, type and extent of dissection in surgery, and use of surgical loupes during surgery were taken into consideration and were assessed in the present study.

Our study observed female predominance, with 80.7% females and only 19.3% males, which is consistent with other studies [4]. The fact that thyroid diseases are more prevalent in the females explains the female predominance in our study. In a previous study, female sex was considered a potential risk factor but in our study, we could not find an association between the female sex and IP [8,16,17]. Using surgical loupes by the surgeon helps better identification of the anatomy. So far, this parameter has not been discussed in the literature based on our best knowledge. In our study, 177 cases were operated by using surgical loupes, despite that IP was found in 42 cases. This suggests that usage of loupes may not have significance in the occurrence of IP, and further research is needed.

Central neck dissection (CND) is the most frequently identifiable risk factor of IP in the related literature [18,19]. In our study, there were limited cases who underwent CND; therefore, an independent association of CND with IP could not be related. However, increased awareness during the dissection of the central compartment is necessary to avoid inadvertent excision of parathyroid tissue. In our study, there was no significant association between the individual risk factors with IP and non-IP cases in comparative analysis similar to few other studies [20,21]. Therefore, as proven by the past studies, knowledge of these factors may help the surgeon to be aware of the risk and be cautious during thyroidectomies.

Limitations of our study include the retrospective nature of the study and the non-consecutive collection of the patients. We did not evaluate the impact of IP on the development of hypoparathyroidism postoperatively. We further recommend routine visualization of parathyroids, implementation of newer technologies like hemostatic devices, magnifying instruments, and fluorescence can prevent incidental removal of parathyroid glands. Thorough examination of surgical specimen and autotransplantation of lower glands may prove to be an effective way to ensure preserved parathyroid function.

## Conclusions

IP is a common finding in thyroidectomy specimen. This often leads to postoperative hypoparathyroidism and hypocalcemia. Deliberate preservation of parathyroids with their native blood supply during total thyroidectomy has direct clinical benefit for patients. Therefore, it is essential to evaluate potential solutions to prevent IP.
